# Greenness Availability and Respiratory Health in a Population of Urbanised Children in North-Western Italy

**DOI:** 10.3390/ijerph17010108

**Published:** 2019-12-22

**Authors:** Giulia Squillacioti, Valeria Bellisario, Stefano Levra, Pavilio Piccioni, Roberto Bono

**Affiliations:** 1 Department of Public Health and Pediatrics, University of Turin, 10126 Turin, Italy; giulia.squillacioti@unito.it (G.S.); valeria.bellisario@unito.it (V.B.); 2 Specialty School in Respiratory Diseases, University of Turin, 10126 Turin, Italy; stefanolevra@gmail.com; 3 Complex Structure of Pneumology, Local Health Authority of the City of Turin, 10126 Turin, Italy; papiccioni@gmail.com

**Keywords:** children health, greenness, respiratory function, environmental primary prevention, asthma

## Abstract

Paediatric Asthma contributes in paediatric global burden of diseases, as the most common chronic disease in children. Children are exposed to many environmental risk-factors, able to determine or worsen respiratory diseases, and contributing to asthma and asthma-like symptoms increases, especially in metropolitan areas. In urban settings, surrounding vegetation (greenness) may provide important benefits to health, including the promotion of physical activity and the mitigation of air and noise pollution. The aim of this study was to investigate the association between greenness and respiratory health. A total of 187 children (10–13 yrs old) were recruited in Turin, the north-western part of Italy. The prevalence of asthma and asthma-like symptoms was calculated from self-reported data collected by SIDRIA questionnaire. Spirometry test was performed to obtain respiratory flow measurements. Greenness was measured at individual level through the Normalised Difference Vegetation Index (NDVI) estimations from remote-sensing images. Higher exposure (3rd tertile vs. 1st tertile) to NDVI was associated to significantly lower ORs for asthma [0.13 CI 95% 0.02–0.7, *p* = 0.019], bronchitis [0.14 CI 95% 0.05–0.45, *p* = 0.001], and current wheezing [0.25 CI 95% 0.09–0.70, *p* = 0.008]. A significative positive association was found between greenness and FEF_25–75_, since children exposed to the 2nd tertile of NDVI reported a significantly decreased FEF_25–75_ compared to those in the 3rd tertile [B: −2.40; C.I.95%: −0.48–0.01; *p* = 0.049]. This cross-sectional study provided additional data on still inconsistent literature referring to respiratory health in children and green spaces, attesting a positive effect of greenness in a specific area of Italy. Further research is still needed.

## 1. Introduction

Paediatric asthma is a widespread condition whose aetiology is multifactorial and may be influenced by a combination of individual and environmental features, such as genetic susceptibility, atopy, and several environmental exposures (e.g., traffic-related pollutants, tobacco smoking exposure, etc.) [1,2]. Asthma contributes in paediatric global burden of diseases, as the most common chronic disease in children [2]. Noticeable changes in the prevalence of asthma were described among countries [1] showing a global prevalence up to a peak of 20% in paediatric population [3]. De Marco [4] reported an increasing trend (+38%) for both asthma and asthma-like symptoms in the Italian population, from 1990 to 2010. In Italy the prevalence of asthma reached 8.6% in children aged 6–7 and 11.4% in adolescents [5]. Concerning respiratory symptoms, current wheezing has been reported as one of the most related to asthma and its prevalence increased in both children (+11.6%) and adolescents (13.7%) [6]. In addition, the assessment of paediatric lung function is used in diagnostic evaluation of respiratory diseases [7] since lung function impairment during childhood may lead to respiratory diseases in adulthood [8] Specifically, a reduced lung functionality in children has been reported as risky for COPD overlap syndrome at 45 years old [8]. At this concern, pulmonary function tests are needful to assess respiratory status at the individual level for investigating the respiratory system and latent abnormalities [9].

Environmental conditions expose children to a variety of factors, which are able to determine or worsen respiratory or allergic diseases [10] contributing to asthma and asthma-like symptom increases, especially in metropolitan areas [11]. Furthermore, as a component of urban environment, green spaces may provide important benefits to health. Higher greenness exposure has been reported as beneficial for several health conditions, such as cardiovascular diseases, adiposity, mental health and birth outcomes [12,13]. Whereas the mechanisms behind greenness exposure and health improvements have not been totally understood yet, it may provide respiratory health improvements by mitigating air and noise pollution [14] or promoting the participation in physical activity [15]. The majority of the studies, investigating respiratory health and the environment, focused on air pollution [16,17,18] and, only recently, increasing attention has been paid to green spaces, whose configuration and composition may differently influence respiratory symptoms and lung function [15]. 

However, the existing results on greenness and respiratory outcomes are still inconsistent [19,20,21]. Some authors reported a positive association between urban vegetation and asthma in children [22], speculating on non-urban vegetation as a potential source for allergens [23,24], which may negatively affect respiratory health. Moreover, living close to the forests has been associated with allergic symptoms [25,26] and greenness was differently associated to allergies, also depending on the study area [27]. On the contrary, several studies stated a protective effect of greenness on respiratory diseases such as asthma. Higher greenness was associated with lower odds ratio of asthma in children with current tobacco smoking exposure and lower risk of [28]. Lovasi et al. reported an inverse association between street trees and prevalence of early childhood asthma [29]. A decreased risk of asthma was observed in children exposed to higher greenness [30], addressing to the “Biodiversity hypothesis”. The proximity to residential greenness was reported as protective for bronchitis and wheezing, in the Mediterranean and Euro-Siberian region, respectively [3]. Finally, even no association has been observed [25,31,32,33]. 

Based on the existing mixed results and their dependency on the geographic area, the aim of this cross-sectional study is to investigate whether there exists an association between green spaces and respiratory health in urban settings, focusing on asthma and asthma-like symptoms in children living in northern Italy.

## 2. Materials and Methods

### 2.1. Sample Population and Ethic Committee Approval

This study is part of a research project funded by the Piedmont Regional Council focusing on the effects of environmental pollution in schoolers that started in 2002 and has followed-up in 2010. Overall, 1005 subjects were enrolled, 573 answered to the follow-up and only 223 accepted to re-perform spirometry. This cross-sectional study involved 187 out of 223 healthy children (10–13 years old) from secondary schools located in Turin, north-western part of Italy. These subjects had validated spirometry, complete questionnaires and provided their home address.

The subjects were recruited at school and participated as volunteers. Each participant gave the assent to participate and the parents or guardians signed a written informed consent to allow their children participation. All healthy volunteers, aged between 10 and 13 years old, who provided the full home address, performed a valid spirometry and reported the total completion of the respiratory questionnaire were included. The study protocol was submitted to the Ethics Committee and was carried out after its approval (Ethics Committee “San Luigi Gonzaga Hospital”, protocol number 826/13/08.) in accordance with the International Ethical Guidelines and Declaration of Helsinki. 

### 2.2. Questionnaire

Demographic and health details were collected in the 2009 through an adapted version of the standardized “SIDRIA” questionnaire [34]. The questionnaire was administered to parents, gathering information about demographic, respiratory symptoms and potential risk factors. In particular, parents answered the following specific questions: “has your child ever had diagnosed asthma?”; “did your child have wheezing during the last 12 years?”; “did your child have wheezing after physical activity during the last 12 months?”;”did your child have cough at night during the last 12 months?”; “did your child have cough excluding when he had cold, during the last 12 months?”; “did your child usually have phlegm excluding when he had cold, during the last 12 months?”; “did your child usually have sneeze excluding when he had cold?”; “has your child ever had allergic colds?”; “has your child ever had skin redness associated to any other symptoms?”; “has your child ever had bronchitis?”; “has your child ever had asthmatic bronchitis?”.

### 2.3. Spirometry

Spirometry was performed in accordance with ATS/ERS standards [35]. Prior to perform the spirometry test, the instrumental calibration was executed through a 3 L syringe. All measurements were carried out early morning at school, concomitant the scholar activities, at the same time of the survey and of the urine collection. Each child underwent spirometry helped and supervised by a team of pneumologists. The spirometry was performed in standing position wearing a nose-clip and breathing a stead-wills spirometer. Children recorded 3–6 maximum expiratory flow-volume curves in the range of 10–15 minutes and those who had only one acceptable measurement were excluded. Exclusion criteria have been mentioned elsewhere [36]. Forced vital capacity (FVC), forced expiratory volume in 1 second (FEV_1_), forced expiratory flow rate 25–75% (FEF_25–75)_ and maximal expiratory flows were measured at 25%, 50% peaks (FEF_25_ and FEF_50_, respectively). FEV_1_/FVC was obtained as ratio and expressed as percentage.

### 2.4. Biological Analyses Cotinine and Creatinine

Each subject provided a sample of urine that was aliquoted and stored at −80 °C until biological analysis. Urinary cotinine was measured as biomarker of exoposure to passive and active tobacco smoking, as described elsewhere [37]. Urinary creatinine was quantified by the kinetic Jaffé method and was used to normalise the excretion rate of cotinine, expressed as ng/mg CREA. 

### 2.5. Green Exposure Assessment

The exposure to urban vegetation was assessed using the Normalised Difference Vegetation Index (NDVI) derived from satellite summer images, cloudy-free and referred to the same year of the sampling procedures (2009). NDVI is a commonly used index in epidemiological studies, able to quantify the vegetated biomass, considering that chlorophyll in healthy vegetation mostly reflects the near-infrared band (NIR) (0.7–1.1 µm) compared to the other wavelengths of the light spectrum and, at the same time, strongly absorbs the visible light (0.4–0.7 µm). NDVI is calculated from the ratio of the difference between the NIR and the RED band to their sum and ranges from –1 to 1, where higher positive values indicate vegetation. 

In this study, NDVI was derived from Landsat 5 satellite images (resolution 30 m × 30 m) and greenness exposure was calculated for all participants within fixed buffers (300 m radius) around their home address, which has been previously geolocalised. 

### 2.6. Other Variables

The Regional Environmental Protection Agency (ARPA) provided the annual average air pollution concentrations. Hence, the levels of air pollutants, namely PM_10_, NO_2_ and NO, were collected from five fixed monitoring stations located within the city boundaries, referred to the year of the sampling procedures and expressed as annual averages (µg/m^3^).

### 2.7. Statistical Analyses

The subjects’ characteristics were summarized depending on the variables of interest: as frequencies and percentages, as appropriate for categorical variables, as means (±SD) or medians (±IQR), as appropriate for continuous variables. The Pearson’s Chi-squared test, ANOVA or Kruskal Wallis, t-test, or Mann-Whitney tests were used to assess the differences among groups, depending on the type and the distribution of the variables. The associations between greenness exposure and respiratory symptoms were assessed through Odds Ratios (ORs), calculated through logistic regression models using respiratory symptoms (0 = not present; 1 = present) as dependent variables and NDVI, divided into tertiles, as the main independent variable. Logistic regression models were adjusted for age, sex, body mass index (BMI) and urinary cotinine levels. Furthermore, Generalised Linear Models were used to test the association between the respiratory flows measured by spirometry and greenness.

The significance level was set ≤5%. Statistical analyses were performed using IBM SPSS® statistic version 26 integrated with R (3.6.1).

## 3. Results

**Figure 1:**
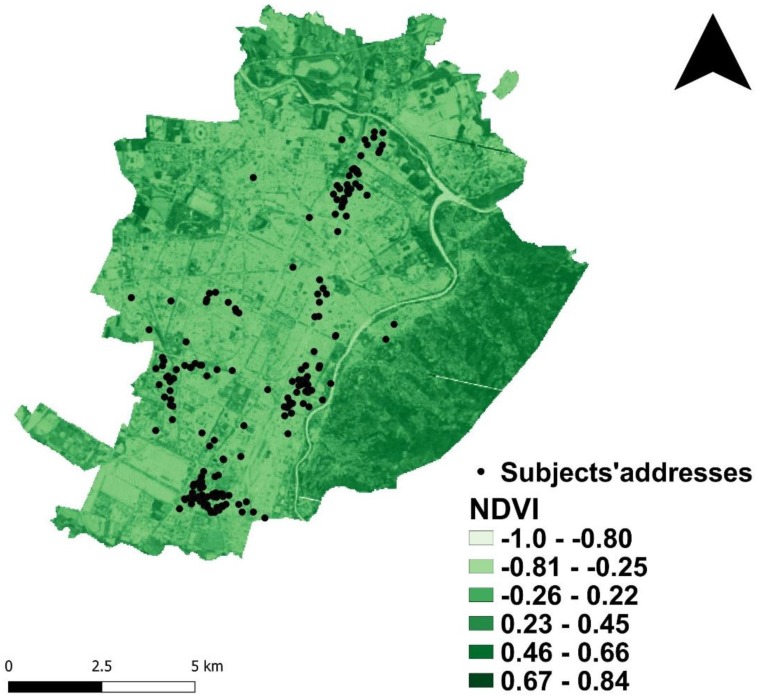
Geolocalised subject’s home addresses on the Normalised Difference Vegetation Index (NDVI) maps within Turin boundaries.

Figure 1 depicts children allocations within Turin boundaries, based on the geolocalised home addresses that their parents provided at the survey time. Table 1 summarises the descriptive statistics of the population sample, splitting data between gender categories. Overall, 187 children aged 10–13 years old, 42% females and 58% males were included in the analysis. The sample is homogeneous for age and anthropometric characteristics (BMI, weight and height), exposure to active/passive tobacco smoking measured, as subjectively by questionnaire (passive cigarettes) as objectively, by urinary cotinine. As shown in Table 1, three out of the whole set of respiratory parameters are significantly different between females and males, these latter show a higher mean value of FEV_1_, FVC and FEFmax (*p* < 0.001, *p* < 0.001 and *p* = 0.010, respectively). The environmental exposures to average annual air pollutants and to urban vegetation, measured by summertime NDVI, are almost the same between genders.

### 3.1. Respiratory Symptoms and Greenness

A set of respiratory symptoms has been investigated by collecting details on the respiratory health through the questionnaire. Based on the cross-sectional design of this study, the prevalence of symptoms and respiratory diseases was calculated for current wheezing (21.4), current wheezing occurring after physical activity (5.9), current coughing during the night (20.3), diagnosed asthma ever (9.6), current cough and phlegm not cold-related (9.6 and 9.1, respectively), current sneezing not cold-related (25.7), allergic colds ever (6.4), skin redness ever (17.1), bronchitis and asthmatic bronchitis ever (19.8 and 6.4, respectively) dealing with the research hypothesis: is greenness associated with respiratory health?

NDVI, originally calculated as continuous variable, was divided into tertiles. At this analysis stage, only first and third tertiles were taken into consideration, including 126 children out of 187, who are supposed to represent the lower exposure (1st tertile) and the higher exposure (3rd tertile) to the vegetated areas of the city. The prevalence of respiratory symptoms and asthma between 1st and 3rd tertiles are almost the same compared to those referred to the whole sample: current wheezing (22.4), current wheezing occurring after physical activity (5.6), current coughing during the night (20.0), diagnosed asthma ever (8.9), current cough and phlegm not cold-related (8.8 both), current sneezing not cold-related (23.3), allergic colds ever (6.4), skin redness ever (17.6), bronchitis and asthmatic bronchitis ever (20.8 and 4.8, respectively). 

**Figure 2:**
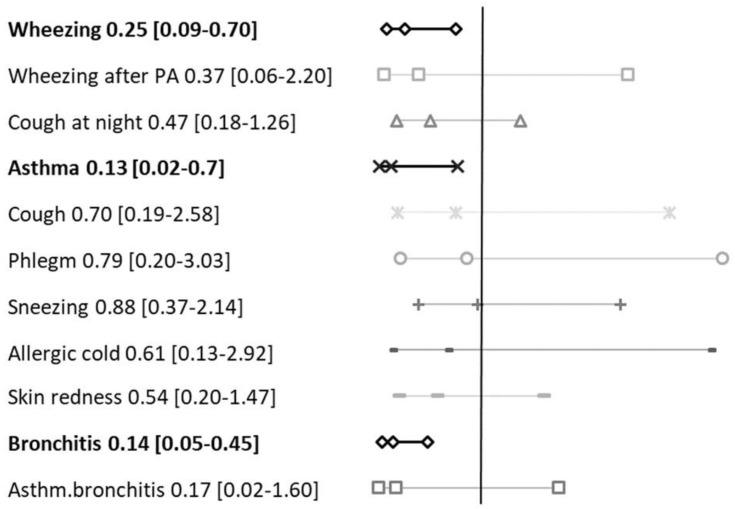
Odds Ratios of symptoms and respiratory disease prevalence referred to children (n = 126) living in more vegetated areas (3rd NDVI tertile) compared to those living in less vegetated areas (1st NDVI tertile).

Odds Ratios (ORs), summarised in Figure 2, were calculated for both symptoms and respiratory diseases in children lying in 1st and 3rd tertiles. Children included in the 3rd tertile reported significantly fewer ORs for current wheezing [0.25 CI 95% 0.09–0.70, *p* = 0.008], asthma [0.13 CI 95% 0.02–0.7, *p* = 0.019] and bronchitis [0.14 CI 95% 0.05–0.45, *p* = 0.001], compared to children from the 1st tertile of NDVI. The other respiratory symptoms were not significantly associated to greenness exposure: wheezing after physical activity [0.37 CI 95% 0.06–2.20, *p* = 0.271]; cough at night [0.47 CI 95% 0.18–1.26, *p* = 0.271]; cough [0.70 CI 95% 0.19–2.58, *p* = 0.583]; phlegm [0.79 CI 95% 0.20–3.03, *p* = 0.727]; sneezing [0.88 CI 95% 0.37–2.14, *p* = 0.786], allergic cold [0.61 CI 95% 0.13–2.92, *p* = 0.537]; skin redness [0.54 CI 95% 0.20–1.47, *p* = 0.226] and asthmatic bronchitis [0.17 CI 95% 0.02–1.60, *p* = 0.120].

Similar results have been obtained between crude and adjusted ORs. Controlling for age, sex, BMI and urinary cotinine levels (showed results) strengthened the association and the significance level for both wheezing [unadjusted OR 0.34 CI 95% 0.4–0.86, *p* = 0.019] and bronchitis [unadjusted OR 0.24 CI 95% 0.09–0.63, *p* = 0.003] and slightly modified the relation with asthma [unadjusted OR 0.13 CI 95% 0.01–0.80, *p* = 0.009]. 

### 3.2. Respiratory Flows and Greenness

The associations between the respiratory flows and greenness were estimated using several Generalised Linear Models (GLMs), testing each respiratory parameter separated and involving the whole sample of children (n = 187). The Table 2 reports results of the GLM specifically used to estimate the association between greenness (expressed as NDVI divided into tertiles) and FEF_25–75_, set as dependent variable, further controlling for some individual variables (sex, age, BMI) and other environmental conditions, such as tobacco smoking exposure and PM_10_ annual average concentrations. The 3rd tertile of NDVI was set as reference category. 

Further results concerning FEF_25_ refer to decreased FEF_25_ in children of the 2nd NDVI tertile compared to those in the 3rd tertile (B: −0.20, C.I.95% −0.40−0.01, *p* = 0.039) and highlight a similar association between greenness and both FEF_25_ and FEF_25–75._

The analyses on other respiratory parameters did not reach the significance level, but showed a statistical tendency for similar trends of the association between NDVI and lung function. In particular, FEV_1_/FVC is diminished in children exposed to lower NDVI values (2nd tertiles) compared to those lying in the 3rd tertile (B = −1.96, C.I.95% −4.22–0.31, *p =* 0.091), controlling for the same set of variables reported in Table 2. Similar results were observed for FEV_1_ (B = −0.11, C.I.95% −0.24–0.02, *p* = 0.097). The exposure to lower NDVI values showed not significant effect on FVC, FEF_50_ and FEFmax (B = −0.07, C.I.95% −0.22–0.9, *p =* 0.393; B = −0.23, C.I.95% −0.51–0.05, *p* = 0.105; B = 0.23, C.I.95% −0.57–0.10, *p* = 0.175, respectively). 

## 4. Discussion

In this study, we observed an association among greenness and some respiratory symptoms and diseases, namely current wheezing, asthma and bronchitis, and an association between greenness and FEF_25–75_ and FEF_25_. In particular, the analysis that involved different levels of exposure to greenness (3rd tertile of NDVI versus 1st tertile) highlighted that children living in greener areas reported less ORs of asthma, current wheezing and bronchitis, even adjusting for age, sex, BMI and cotinine. On the other hand, by analysing the association between greenness and lung function, children living in less vegetated areas (2nd tertile vs 3rd tertile of NDVI) showed a decrease in FEF_25–75_, even controlling for BMI, sex, cigarettes/day and annual average PM_10_ air concentrations.

Our results are in line with some of already published findings. For example, residential surrounding greenness has been previously reported as a protective factor for lifetime wheezing in a population of Mexican children living in Chicago [28]. Tischer et al. [3] observed that children living in the Euro-Siberian area in Spain have reduced risk of wheezing. At this concern, Turin is placed in a different bio-geographic area, namely Continental, which shares only few characteristics with the Euro-Siberian such as the humid climate and the peak in leaves biomass during the summer [38]. Urban areas with greater street tree density were associated with a lower prevalence of asthma in American children, investigated through an ecological approach in New York city [29]. A decreased risk of asthma was also reported in association with an increase in NDVI value at individual level [30]. Moreover, children living in more vegetated areas in New Zeeland showed a lower risk of having asthma [39]. Other authors reported no association between greenness and asthma. A cross-sectional study in Spain found that greenness was not associated with current asthma in children [25] or with asthma prevalence in unadjusted analyses [32]. 

Concerning the odds for bronchitis in childhood, only one study stated a significantly inverse association of greenness and bronchitis [3]. However, Tischer observed that higher NDVI levels were associated to lower risk of bronchitis only in Spanish children living in the Mediterranean area of the country. The widely held theory that could explain how greenness can improve respiratory health is related to its capacity to enhance environmental biodiversity [40] and reduce exposure to air pollution or even promoting physical activity and prevent overweight and obesity.

On the contrary, our results are in contrast with other studies, which reported that greenness is positively associated with asthma and allergic conditions. Andrusaityte stated that a greater amount of vegetation is associated with higher relative risk of asthma in Lithuanian children [22]. Living in proximity of the parks was associated with a higher relative prevalence of current asthma in children [25,41]. Many authors suggested few mechanisms by which greenness can act as risk factor in worsening respiratory condition, for example by releasing pollens and fungal spores in the environment [24,42,43], or even increasing the exposure to pesticides and fertilisers [44].

To the best of our knowledge, no already published works investigated the association between greenness and spirometry parameters in children, except for conference abstracts and a study that measured lung functionality through the Forced Oscillation Technique [45].

The fact that NDVI is significantly associated only with FEF_25–75_ and FEF_25_ may indicate a particular susceptibility of small airways under the effect of greenness.

Even if, no statistical significance level was reached, both FEV_1_ and FEV_1_/FVC are lower in adolescents exposed to lower NDVI values. Therefore, our data suggests that greenness may influence respiratory function as a whole. From this point of view, it would be interesting to undertake future studies using tests that, while analysing the entire respiratory system, allow to specifically focus on the small airways, such as the Forced Oscillation Technique, which seems even more sensitive in detecting the early damage of the airway [46].

## 5. Strengths and Limitations

The cross-sectional design of the present study is the main limitation that did not allow us to assess the causation direction in general and with clear consequences in the ORs interpretation. Another limitation is that we measured only the exposure to the quantity of green spaces (NDVI) without specifying the type of vegetation and that the sample size might be considered relatively small to investigate all around the lung function.

As remarkable strength, this study is the first that investigated the association between green spaces and respiratory health in this specific geographic area, providing specific data, which respond to the important issue of the geographic variability of these associations [23,27,41,47].

## 6. Conclusions

Our results support that greenness has an association with respiratory health in children and provide information about a specific geographic area of Italy. Data are in line with some author’s results and in contrast with others, indicating that greenness and respiratory health is still poorly understood. Further research is needed to understand the specific mechanism by which urban vegetation may interact with the respiratory health and to enhance the exposure assessment by multi-location assessment and the type characterisation.

## Figures and Tables

**Table 1:** Demographics and general characteristic of the whole sample.

Sex n (%)	Female 79 (42)	Male 108 (58)	*p*-Value	All n = 187
**Age** (yrs)	11.5 ± 0.7	11.6 ± 0.9	0.465	11.5 ± 0.8
**BMI** (kg/m^2^)	18.6 ± 3.2	19.7 ± 3.8	0.095	19.3 ± 3.6
**Weight** (kg)	41.6 ± 9.7	45.3 ± 11.9	0.077	43.8 ± 11.2
**Height** (cm)	148.8 ± 8.5	150.8 ± 9.4	0.205	150 ± 9.1
**Passive cigarettes** (n/day)	5.1 ± 1.2	4.8 ± 1.3	0.266	4.9 ±1.3
**Cotinine** (ng/mgCREA)	0.38 ± 1.5	0.36 ± 0.86	0.870	0.61 ± 0.59
**FEV_1_** (L)	2.2 ± 0.4	2.4 ± 0.5	**<0.001**	2.3 ± 0.5
**FVC** (L)	2.5 ± 0.5	2.9 ± 0.6	**<0.001**	2.7 ± 0.6
**FEV_1_/FVC** (%)	86.1 ± 7.3	85.7 ± 5.7	0.085	85.9 ± 6.4
**FEF_25_** (L/sec)	1.4 ± 0.5	1.6 ± 0.6	0.210	1.5 ± 0.6
**FEF_25–75_** (L/sec)	2.5 ± 0.7	2.7 ± 0.8	0.201	2.6 ± 0.7
**FEF_50_** (L/sec)	2.9 ± 0.8	3.1 ± 0.8	0.089	3.0 ± 0.8
**FEF_max_** (L/sec)	4.3 ± 1.0	4.7 ± 1.0	**0.010**	4.5 ± 1.0
**PM_10_** (µg/m^3^)	48.7 ± 6.5	48.9 ± 7.0	0.934	48.8 ± 6.8
**NO_2_** (µg/m^3^)	55.8 ± 14.2	55.4 ± 14.8	0.934	55.6 ± 14.5
**NO** (µg/m^3^)	45.3 ± 15.1	45.5 ± 16.2	0.934	45.4 ± 15.7
**NDVI**	0.25 ± 0.07	0.26 ± 0.07	0.445	0.25 ± 0.07

Table footer: significant *p*-value bolded in the table.

**Table 2:** Generalised Linear Model results, estimating the association between NDVI dividend into tertiles (exposure variable) and forced expiratory flow rate 25–75% (FEF_25–75_) (outcome variable) in the whole sample (n = 187). The 3rd NDVI tertile was set as reference category.

Variables	B	C.I. 95%	*p*-Value
Intercept	2.11	1.32–2.90	**<0.001**
BMI	0.01	−0.02–0.04	0.370
Age	**0.30**	0.18–0.42	**<0.001**
Sex	0.12	−0.08–0.31	0.245
PM_10_	0.01	−0.01–0.03	0.073
Cigarettes/day	−0.21	−0.01–0.06	0.595
NDVI 1st tertile	0.06	−0.19–0.30	0.640
NDVI 2nd tertile	**−2.40**	−0.48–0.01	**0.049**

Table footer: significant *p*-value bolded in the table.
